# Bioinformatics-Assisted Discovery of Antioxidant Cyclic Peptides from Corn Gluten Meal

**DOI:** 10.3390/foods14101709

**Published:** 2025-05-12

**Authors:** Hongcheng Liu, Tong Sun, He Gao, Xiaolong Liu, Shanshan Zhang, Tingting Liu, Dawei Wang, Hongxiu Fan, Yanrong Zhang

**Affiliations:** 1 School of Food Science and Engineering, Jilin Agricultural University, Changchun 130118, China; liuhongcheng@jlau.edu.cn (H.L.); suntong08051320@163.com (T.S.); 13756806278@163.com (H.G.); zhangshanshan@jlau.edu.cn (S.Z.); liutingting@jlau.edu.cn (T.L.); wangdawei@jlau.edu.cn (D.W.); 2 Scientific Research Base of Edible Mushroom Processing Technology Integration of Ministry of Agriculture and Rural Affairs, Changchun 130118, China; 15030377650@163.com; 3 Engineering Research Center of Grain Deep-Processing and High-Efficiency Utilization of Jilin Province, Changchun 130118, China; 4 Key Laboratory of Technological Innovations for Grain Deep-Processing and High-Efficiency Utilization of By-Products of Jilin Province, Changchun 130118, China

**Keywords:** antioxidant cyclic peptide, bioinformatics, structural characterization, molecular docking

## Abstract

Using a multidisciplinary approach, this paper was designed to prepare, identify, and characterize novel maize antioxidant cyclic peptides from protein hydrolysate of corn gluten meal (CGM). A bioinformatics approach was used to identify the best protease, and the results showed that papain+subtilisin was most likely to produce antioxidant cyclic peptides. The result of the enzymatic hydrolysis validation experiment showed that hydrolysate by papain+subtilisin yielded the highest concentration of cyclic peptide (67.14 ± 1.88%) and remarkable DPPH, ABTS, and hydroxyl radical scavenging rates (81.06 ± 2.23%, 82.82 ± 1.83%, and 47.44 ± 2.43%, respectively) compared to other hydrolysates. Eleven antioxidant cyclic peptides were identified in the protein hydrolysate of CGM through sequential purification and mass spectrometry analysis. The results of molecular docking analysis indicated that the cyclic peptides can form stable hydrogen bonds and hydrophobic interactions with the key amino acid residues of Kelch-like ECH-associated protein 1 (Keap1). Cyclic peptides may regulate the Keap1-Nrf2 pathway by occupying the Kelch domain of Keap1, inhibiting the ubiquitination degradation of Nrf2 (nuclear factor erythroid 2-related factor 2), thereby stabilizing the Nrf2 protein and activating the antioxidant gene network. This study underlined the bioinformatics approach for antioxidant cyclic peptide discovery, which is time- and cost-effective and promotes new cyclic peptide drugs or functional food development.

## 1. Introduction

Antioxidant peptides derived from food resources are in wide demand in the nutraceuticals, food, and cosmetics industries, as they are safe and highly efficient [1]. Peptides can be categorized into straight-chain peptides and cyclic peptides based on the structure of the peptide bond. Traditional linear peptides are less stable and prone to degradation under thermal or enzymatic conditions [2]. Compared to linear peptides, cyclic peptides have a relatively stable conformation, in vivo metabolic stability, cell membrane permeability, and better resistance to enzymatic degradation [3]. The preparation methods for cyclic peptides include fermentation, enzymatic hydrolysis, chemical synthesis and direct extraction. Protein enzymolysis and fermentation are promising methods for cyclic peptide generation because of their mild condition, high safety, low cost, and low chemical pollution [4]. Recently, various cyclic peptides were shown in protein hydrolysates and fermentation broths. It was reported that cyclic peptide structures could be synthesized by cyclization of the N/C termini of the linear peptides (head to tail) via an amide bond. The transaction of linear peptides to cyclic peptides usually happens under high temperatures, polar solutions, or higher pH values [5]. Routhu et al. isolated and characterized a novel antifungal cyclic peptide from marine isolated Neobacillus drentensis [6]. Zhao et al. extracted five cyclic peptides, including cyclic pentapeptides, hexapeptides, and heptapeptides, from fermented fig [7]. They proved that these five cyclic peptides have good antioxidant and immunomodulatory effects. Our previous study reported Cyclo(His-Pro) isolated from the protein hydrolysate of corn. This cyclic dipeptide has hypoglycemic and antioxidant activities [8].

Corn gluten meal (CGM) is a by-product of wet corn starch production, with a protein content of about 60%. China is estimated to produce approximately 288 megatons of maize annually, and 17% of maize was utilized for starch production, generating an estimated 3.2 megatons of CGM. However, CGM is often used as feed or discarded due to its imbalanced amino acids, bitter taste, and poor solubility in water [9]. Previous research has demonstrated that CGM is a good raw material for preparing bioactive peptides, especially peptides with antioxidant activity, because of its unique amino acid composition [10]. The protein of CGM is mainly composed of hydrophobic amino acids (such as Phe, Leu, Pro, Ala, Ile, etc.) that are strongly associated with the antioxidant properties of peptides [11,12]. Moreover, many scholars have shown that amino acids that have a turn structure or restricted conformation (such as Pro) or small amino acids that have less steric hindrance (such as Ala, Gly) are often found in cyclic peptides, indicating that linear peptides that contain these amino acids are prone to forming cyclic peptides through head-to-tail cyclization [13]. Since the proteins in CGM are rich in Pro and Ala, it can be inferred that CGM has excellent potential for preparing cyclic peptides with antioxidant activities.

Nowadays, conventional approaches for preparing and identifying bioactive peptides have been found to be lengthy, time-consuming, and laborious. Thus, a new bioinformatics approach involving analytical in silico tools has emerged as an invaluable complement to the research of bioactive peptides [14]. This approach could select the appropriate enzyme for the hydrolysis of protein by narrowing down the number of enzyme combinations and rapidly screening and characterizing potential functional peptides, thereby reducing costs, improving efficiency, and accelerating study [15]. Generally, the bioinformatics approach comprises several steps: parent protein identification or searching parent protein databases, enzymolysis simulation, and bioactivity prediction through database comparison [16]. In addition, the bioinformatics approach can be combined with conventional approaches, thus improving the efficiency of functional peptide identification processes [17]. Many novel functional peptides with ACE inhibitory, antioxidant, anti-diabetic, and antimicrobial activities have recently been discovered from laver, curcumin, pinto bean, and sorghum bicolor using bioinformatics approaches [18,19,20,21,22]. However, no reports apply the bioinformatics approach to discovering antioxidant cyclic peptides.

Keap1 protein plays a core regulatory role in the antioxidant signaling pathway. It can promote the ubiquitination and degradation of Nrf2 by binding to the nuclear transcription factor Nrf2, thereby inhibiting the expression of antioxidant enzyme genes [23]. In addition, there are clear binding sites on the Kelch domain of Keap1. Antioxidant peptides can compete for binding sites, block the interaction between Keap1 and Nrf2, release Nrf2 into the cell nucleus, and activate the expression of downstream antioxidant enzyme genes, thereby enhancing the antioxidant capacity of cells [24]. Federica Tonolo et al. [25] extracted and purified antioxidant peptides from fermented soy products, and used molecular docking methods to predict their ability to interact with Keap1. Therefore, this paper uses Keap1 as a receptor protein for molecular docking with cyclic peptides to reveal the antioxidant mechanism of cyclic peptides at the molecular level and provide a theoretical basis for the development of new natural antioxidants.

It is known that cyclic peptides could be produced from fermentation broths and protein hydrolysates. However, studies exploring bioactive cyclic peptides from food-derived protein sources are scarce. Cyclic peptides are believed to be essential in advancing functional food and biomedicine, as they offer unique structural and functional advantages over linear peptides. Thus, obtaining more information about cyclic peptides’ structure and functional properties is essential. This study used bioinformatics and mass spectrometry identification methods to explore new antioxidant cyclic peptides in CGM enzymatic protein. The bioinformatics approach was applied to predict potential linear peptide precursors and to guide the enzyme selection or enzyme combinations to optimize the enzymolysis of substrate proteins [26]. Moreover, cyclic peptide sequences in the hydrolysate were identified through tandem mass spectrometry technology. Finally, the molecular docking technique was used to assess the interaction between cyclic peptides and Keap1, providing insights into their potential mechanism of action. This study could have significant implications for researching and developing food-derived bioactive cyclic peptides.

## 2. Materials and Methods

### 2.1. Materials and Chemicals

The CGM was from Gongzhuling Huanglong Corn Co., Ltd. (Changchun, China). The subtilisin (560 U/mg) was from Novozymes Ltd. (Bagsvaerd, Denmark). The stem bromelain (300 U/mg) was from Shanghai Yuanye Biotechnology Co., Ltd. (Shanghai, China). The papain (900 U/mg) was from Ruiyong Technology Co., Ltd. (Shanghai, China). All other analytical-grade chemicals were from Sinopharm Chemical Reagent Co., Ltd. (Beijing, China).

### 2.2. In Silico Digestion of Protein of CGM and Enzyme Selection

The bioinformatics tool BIOPEP (https://biochemia.uwm.edu.pl/biopep-uwm/, accessed on 14 June 2023) was used to perform the in silico digestion of protein sequences of CGM. Six kinds of zeins, including sorghum bicolor hypothetical protein (gi|241917067), 22 kDa α-zein (gi|22179), z1B_6 precursor (gi|162459436), z1A1_2precursor (gi|162459254), β-zein precursor (gi|162463731), and γ-zein precursor (gi|821325083), and 2 kinds of glutelins, including globulin-1 S (gi|162463479) and legumin 1 (gi|16305144), from CGM were used in the in silico digestion. Our previous studies proved these proteins to be abundant in CGM protein. Corn protein sequences were collected from the NCBI database (https://www.ncbi.nlm.nih.gov/, accessed on 21 May 2023) and stored in FASTA format after verification by comparing sequence identity, sequence accession number, species name, and gene name. Papain (EC 3.4.22.2), stem bromelain (EC 3.4.22.32), and subtilisin (EC 3.4.21.62) were selected from the BIOPEP database. The protocol of in silico hydrolysis was as follows: papain, stem bromelain, subtilisin, papain+stem bromelain, papain+subtilisin, and stem bromelain+subtilisin. Using BIOPEP-UWM, fragments with antioxidant activity generated by each enzyme or enzyme combination were predicted. In addition, referring to the study by Tang et al., 2000 [27], the number of easily cyclized fragments was predicted based on the easy cyclization properties of each peptide after virtual enzymatic hydrolysis. Peptides with easy cyclization properties mainly include peptides with reactive groups such as Cys, Lys, and Glu on the side chains; short peptides with N-terminal amino groups and C-terminal carboxyl groups; and peptides with special conformations, such as Pro and Gly.

### 2.3. Pretreatment of CGM

CGM was defatted using a supercritical CO_2_ device according to the method of Liu et al., with slight modifications. The CGM was degreased at 25 MPa and 45 °C for 4 h [28]. Referring to the method of Wijethunga et al. [29], defatted corn flour (CGM) was treated for starch removal. A total of 60 g defatted CGM was weighed, a mixed solution at a solid–liquid ratio of 1:3 was prepared, the pH was adjusted to 6.5, and thermostable α-amylase was added and hydrolyzed for 1.5 h in a 90 °C water bath. After the reaction was completed, the solution was heated at 100 °C to inactivate the enzyme for 15 min and centrifuged to separate the enzymatic hydrolysis product, and the precipitate was collected after washing and dried for later use.

### 2.4. Hydrolysis of Protein of CGM and Cyclization of Linear Peptide Precursors

Enzymatic hydrolysis of the protein of CGM was conducted using the three proteases. The pretreated CGM was mixed with deionized water to prepare the substrate at 1:40 (*w/v*). The proteolysis conditions of the single enzyme and enzyme combinations are shown in Table 1. The hydrolysis was stopped by heating in a water bath at 95 °C for 15 min. After the hydrolyzate was centrifuged at 3800 r/min for 15 min, three volumes of ethanol were added to the supernatant and allowed to stand for 3 h to remove the protein. Then, the supernatant was lyophilized and kept for subsequent experiments.

The cyclization of linear peptide precursors was performed with slight modifications based on a previously reported method [30]. To the 70% (*v/v*) ethanol solution (100 mL), enzymatic hydrolysate (1 g) was added and the pH of the solution was adjusted to 8.0 with KHCO_3_. Then, the solution was subjected to reflux and stirred at a temperature of 60 °C. During the reaction, the C-terminal residue of linear peptide precursors was converted into highly reactive esters, facilitating the formation of an amide bond between the N- and C-terminal residues, thereby generating cyclic peptides [31]. After the cyclization reaction was completed, the enzymatic hydrolyzate was filtered, concentrated, and freeze-dried.

### 2.5. Determination of Antioxidant Activity

#### 2.5.1. DPPH Radical Scavenging Activity

According to previous reports [32], corn antioxidant cyclic peptides’ DPPH radical scavenging activity was detected. The absorbance of the samples was recorded at 517 nm. The DPPH free radical scavenging activity was calculated using the following formula:

(1) $$DPPH\;radical\;scavenging\;rate\;\left(\%\right)=\frac{\left(A_{0}-A_{1}\right)}{A_{0}}\times 100\%$$

In Formula (1), A_0_ is the absorbance of the control and A_1_ is the absorbance of the sample.

#### 2.5.2. ABTS Radical Scavenging Activity

ABTS radical scavenging activity of antioxidant cyclic peptide was determined as described previously [33]. A total of 0.04 mL (8 mg/mL) of peptide sample was added to 2 mL of diluted ABTS radical solution and the mixture was incubated for 6 min. Subsequently, the absorbance of the mixed solution was measured at a wavelength of 734 nm. The ABTS radical scavenging rate was calculated using the following formula:

(2) $$ABTS\;radical\;scavenging\;rate\;\left(\%\right)=\frac{\left(A_{0}-A_{1}\right)}{A_{0}}\times 100\%$$

In Formula (2), A_1_ is the absorbance in the presence of the sample, and A_0_ is the absorbance of the control reaction.

#### 2.5.3. Hydroxyl Radical Scavenging Activity

The hydroxyl radical scavenging ability of antioxidant cyclic peptides was determined using Chen et al.’s method [34]. The absorbance was measured at 510 nm. The hydroxyl radical scavenging rate was calculated using the following formula:

(3) $$Hydroxyl\;radical\;scavenging\;rate\;\left(\%\right)=\frac{\left(A_{0}-A_{1}\right)}{A_{0}}\times 100\%$$

In Formula (3), A_0_ and A_1_ indicated the absorbance of the sample and control group, respectively.

### 2.6. Qualitative and Quantitative TLC Analyses of Cyclic Peptides

Analytical TLC plates were used to analyze cyclic peptides in the hydrolysate (10 × 2.5 cm, 0.3 mm) [35]. Hydrolysate samples were dissolved in methanol (2 mg/mL) and spotted separately on two analytical TLC plates. The two plates were developed in a glass chamber using a chloroform–methanol solvent system (85:15, *v/v*). After development, one of the TLC plates was sprayed with ninhydrin solution to visualize the spots of linear peptides and proteins. Cyclic peptides cannot be visualized under this condition because they do not have a free amino group at the backbone. According to the previous method, the other TLC plate was hydrolyzed with HCl at 105 °C for 1 h in a sealed glass chamber. The acid-hydrolyzed TLC plate was then sprayed with ninhydrin to visualize the spots of cyclic peptides present in the sample. HCl hydrolysis could lead to the breakage of the ring structure of cyclic peptides, exposing the free amino group. Then, the acid hydrolysis breaks the cyclic structure, exposing amino groups for ninhydrin detection in the ninhydrin test [36]. The retention factor (R_f_) for each spot was determined.

The preparative TLC plate (10 × 2.5 cm, 1 mm) was used for cyclic peptide quantitative analysis of the hydrolysate. The sample solution was spotted onto a preparative TLC plate and developed in the solvent system of chloroform–methanol (85:15, *v/v*). The produced spots were checked by iodine vapor and confirmed using R_f_ values. Silica containing the cyclic peptides was scraped from the TLC plates, extracted into methanol, and filtered. The hydrolysate and cyclic peptide extract’s peptide contents were estimated using the biuret method. The calculation formula for cyclic peptide yield was as follows:

(4) $$Cyclic\;peptide\;yield=\frac{A_{1}}{A_{2}}\times 100\left(\%\right)$$

In Equation (3): A_1_ indicates the peptide content of the hydrolysate that was spotted on the TLC plate (mg) and A_2_ indicates the peptide content in the enzymatic hydrolysate (mg).

### 2.7. Separation and Purification of the Antioxidant Cyclic Peptide from Protein Hydrolysate of CGM

#### 2.7.1. Silica Gel Column Chromatography

A total of 50 g of silica gel (300 mesh) activated at 105 °C for 2 h was loaded into a 40 × 1.6 cm silica gel column. A total of 0.2 g of hydrolysate sample was subjected directly to the column and separated with a step gradient elution of methanol chloroform (2:8, 3:7, 4:6, 5:5, *v/v*) to yield four fractions (F1–F4). Each fraction was subjected to TLC analysis, and the antioxidant activity of each fraction was evaluated.

#### 2.7.2. Semi-Preparative Reversed-Phase High-Performance Liquid Chromatography (RP-HPLC)

Using a semi-preparative RP-HPLC system (Waters 1525-2489-WFCIII), the fractions obtained through silica gel column chromatography were further separated on a SunFire C18 OBD™ (Waters, Milford, MA, USA) prep column (10 mm × 150 mm, 5 μm particle size) at a flow rate of 1 mL/min. A gradient elution mode was employed with acetonitrile and water as the mobile phase, programmed to change from 10% to 80% throughout 0 to 30 min. A UV-visible detector was used to monitor all fractions at a wavelength of 220 nm, and the DPPH, ABTS, and hydroxyl radical scavenging activity assays assessed the antioxidant activity of each fraction.

### 2.8. Structural Characterization of Cyclic Peptides

#### 2.8.1. UPLC/MS and MS/MS Analysis

The amino acid sequence of the purified cyclic peptide fractions with the higher antioxidant activity was determined using UPLC-Q-TOF-MS and Orbitrap-MS/MS systems according to a previously developed method in our laboratory. Separation of cyclic peptides was achieved with Waters ACQUITYUPLC using a BEH C18 column. A mobile phase consisting of acetonitrile (A) and ultrapure water (B) in a gradient elution mode (0–20 min, 20–40% A; 20–30 min, 40% A) was used. MS and MS/MS experiments characterized the cyclic peptide sequence. In the MS experiment, a Q-TOF-MS system (Synapt G2-SI, Waters, Milford, MA, USA) was equipped with an electrospray ionization source and operated in positive ionization mode. The ion source and desolvation gas temperatures were maintained at 120 °C and 350 °C, respectively. The full MS scan range was 100–1500 m/z mass.

The MS/MS analysis was performed using an Orbitrap Elite mass spectrometer (Thermo LTQ, Thermo Fisher, Darmstadt, Germany) equipped with a peristaltic pump and an electrospray ionization source. The desolvent gas flow rate was 0.5 L/min. The capillary voltage was 5 KV. The sample solution was sent to the nebulizer at a 5 µL/min flow rate. According to the method of Liu et al. [28], the cyclic peptide sequence was determined based on the mass difference between adjacent “y”- or “b”-type ions in the MS/MS spectrum.

#### 2.8.2. Detection of the Cyclization Sites of Cyclic Peptides

The cyclization sites of cyclic peptides cannot be detected directly using MS/MS analysis. Thus, we identified the linear peptide precursors in the protein hydrolysate of CGM using LC-MS/MS analysis. The analysis conditions were as follows: Chromatography was performed on a reversed-phase C18 column (2.1 × 100 mm, 2.7 μm). The binary mobile phase consisted of phase A (ultrapure water—0.1% formic acid) and phase B (acetonitrile—0.1% formic acid), and the gradient elution was as follows: 8–30% B from 6 to 40 min, 30–80% B from 40 to 48 min. A 0.4 μL/min flow rate was used, with an injection volume of 5 μL. The mass spectrometer was operated in positive mode with an ion source voltage of 1800 V and a capillary temperature of 360 °C. The scan mode was a full scan with a spectral range of 350–2000 (m/z) and a scan resolution of 70,000. The product ion scan range began at 100 m/z, with CID as the activation type and a secondary collision energy set to 28% for MS/MS analysis. The mass spectra data were processed using Mascot Software 2.2. The cyclization sites of the antioxidant cyclic peptides can be determined by comparing the structures of antioxidant cyclic peptides and their linear peptide precursors.

### 2.9. Molecular Docking Analysis

The molecular docking analysis was performed using DiscoveryStudio 2.5 software according to the method of Cai et al. [37]. The crystal structure of the receptor protein Keap1 (PDB: 2FLU) was downloaded from the PDB database. Chem Draw 3D 19.0 was used to modify the protein structure, remove water molecules and small molecules in the protein structure, add hydrogen atoms to the receptor protein, and minimize the energy of the receptor protein. After checking the integrity of the protein structure, the receptor protein was saved in pdb format. The three-dimensional structure of the small molecule peptide was also drawn using ChemDraw3D 19.0. DiscoveryStudio software was used to first remove water molecules and small molecules in the ligand molecule, perform hydrogenation treatment, check the rotation bonds, and set the parameters (network center coordinates are x = −10.7, y = −20.0, z = −19.5, and the box size is 60 Å × 60 Å × 60 Å) for molecular docking. The molecular docking results are expressed as CDOCKER energy (≤50 Kcal/mol).

### 2.10. Data Statistical Analysis

Excel 2021 and SPSS 25 were used for data processing, Origin 2021 was used for image drawing, and all experiments were performed at least 3 times in parallel.

## 3. Results

### 3.1. Selection of Enzyme or Enzyme Combination Using a Bioinformatics Approach

**Figure 1:**
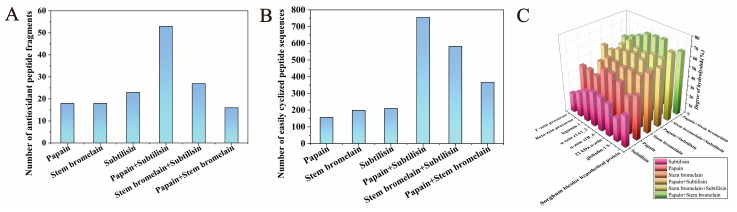
Number of peptides with antioxidant activity (**A**), number of peptides that are easy to cyclize (**B**), and the DH value (**C**) produced by the in silico proteolysis of proteins of CGM by different enzymes and combinations.

Since zein and glutelin are the main proteins in CGM, in this study, we chose eight zein and glutelin sequences as parent proteins for in silico proteolysis. Three enzymes (papain, stem bromelain, and subtilisin) were selected from the online BIOPEP-UWM platform, and they were used separately and in combination in the in silico proteolysis of proteins in CGM. Figure 1C shows the DH (degree of hydrolysis) of the hydrolysate from the in silico hydrolysis. It can be seen that compound-enzyme treatment, including papain+stem bromelain, stem bromelain+subtilisin, and papain+subtilisin, exhibited higher DH values compared with their corresponding single enzyme. This indicated that compound enzymes exhibited more favorable hydrolysis conditions and released diverse peptides. The BIOPEP-UWM platform was employed to predict the biological activities of peptides generated by various enzymes and to identify peptide fragments with antioxidant properties. Figure 1A illustrates the number of peptide fragments with antioxidant activity released by either a single or a compound enzyme. It can be observed that compared with a single enzyme, compound-enzyme treatment released higher numbers of antioxidant peptides. Papain+subtilisin released the highest number of antioxidant peptide fragments. This indicated that compared to single-enzymatic hydrolysis, combined enzymatic hydrolysis is more effective and increases the antioxidant activity of hydrolysate. This is consistent with the results of other studies [38]. Wu et al. reported that combinatorial enzymatic hydrolysis could make obtaining various peptides easier, which may increase the biological activity of hydrolysates [39].

It was reported that cyclic peptide structures could be synthesized by cyclization of the N/C termini of the linear peptides (head to tail) via an amide bond [40]. Some structural factors of linear peptides, including the type and position of amino acids within the sequence, can influence their cyclization efficiency. For instance, the presence of small amino acids (such as Gly, Ala, Ser, etc.) in the terminal region of the peptide sequences and the presence of conformationally restricted amino acids (such as Pro and Gly) in the peptide sequence has been of importance for the cyclization of linear peptide [41]. Moreover, cyclization is regarded as the most probable modification process for dipeptides containing Leu, His, Ile, Pro, Ala, Gly, Tyr, Phe, Val, Met, and Gln [42]. These structural characteristics of cyclic peptides could be considered essential parameters for the virtual screening of peptide sequences that are easy to cyclize. Based on the above information, the number of easy peptide fragments to cyclize was calculated for each single- or compound-enzyme hydrolysis. In silico hydrolysis using compound enzymes was found to enhance the number of antioxidant peptides significantly and increase the number of peptides that are easy to cyclize. The in silico hydrolysis with papain+subtilisin released the highest number of easily cyclized peptides (Figure 1B).

Therefore, considering the antioxidant activity and the number of peptides prone to cyclization, papain and subtilisin were selected to hydrolyze the protein of CGM to produce cyclic peptides with antioxidant properties.

**Figure 2:**
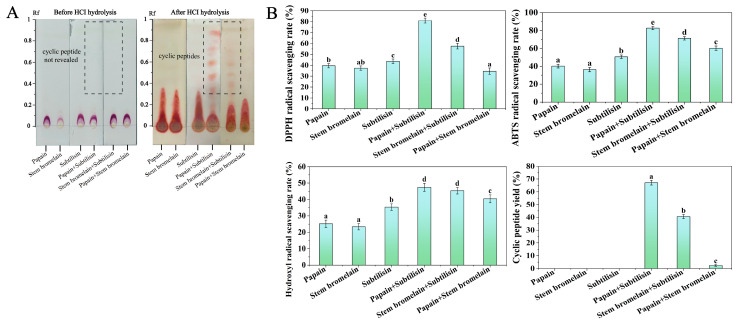
TLC chromatograms (**A**) and scavenging rates of DPPH, ABTS, and hydroxyl radicals and cyclic peptide yield (**B**) of protein hydrolysate of CGM produced by different enzyme combinations. Different lowercase letters (a–e) on the bars mean significant difference (*p* < 0.05).

In order to validate the optimal enzyme combination selected by in silico hydrolysis, the pretreated CGM was hydrolyzed by papain, stem bromelain, and subtilisin. Figure 2A shows the TLC chromatograms of various enzymatic hydrolysates. Prior to HCl hydrolysis, the TLC chromatograms of all hydrolysates displayed spots of linear peptides or proteins around the starting point. The cyclic peptides in the hydrolysates cannot be visualized prior to HCl hydrolysis because they lack a free N-terminus. After HCl hydrolysis, the TLC chromatograms of the hydrolysates treated by papain+subtilisin and stem bromelain+subtilisin showed spots of cyclic peptide at an R_f_ of between 0.45 and 0.9 (as shown in Figure 2A). However, on the TLC chromatograms of other hydrolysates, the spots of cyclic peptides were not observed, indicating that the cyclic peptides were in small amounts or were not produced in other hydrolysates. The preparative TLC method was used for the quantitative analysis of cyclic peptides in the hydrolysates, and the cyclic peptide yields are presented in Figure 2B. It can be seen that the hydrolysate by papain+subtilisin had the highest cyclic peptide yield (67.14 ± 1.88%), followed by stem bromelain+subtilisin (40.65 ± 1.66%). The antioxidant capacity of hydrolysates was evaluated by measuring the scavenging rates of DPPH, ABTS, and hydroxyl radicals, and the results are shown in Figure 2B. The antioxidant activity of cyclopeptides was determined at a concentration of 2.0 mg/mL. Compared with single-enzyme hydrolysis, the cyclopeptide yield and DPPH, ABTS, and hydroxyl radical scavenging rates of composite-enzyme hydrolysis were higher. In addition, hydrolysate treated by papain+subtilisin exhibited the highest DPPH, ABTS, and hydroxyl radicals scavenging rates (81.06 ± 2.23%, 82.82 ± 1.83%, 47.44 ± 2.43%). The enzymatic hydrolysis results revealed that the hydrolysate produced using papain+subtilisin contained the highest amount of cyclic peptides and exhibited superior antioxidant activity. Therefore, papain and subtilisin appeared to be the most suitable enzymes for hydrolyzing the protein of CGM to produce cyclic peptides with antioxidant properties, consistent with the result of in silico proteolysis simulations.

Although the bioinformatic approach is a promising way to develop bioactive peptides, some challenges remain, such as enzymatic hydrolysis parameters (enzyme addition, incubation time, pH, temperature) not being considered. Tools are typically effective for predicting a single type of bioactivity for one specific protein substrate when there are limited substrate protein data in the database. At the same time, virtual enzymatic hydrolysis usually focuses on linear sequence analysis and ignores its three-dimensional structure and dynamic interactions. However, the activity of a peptide may depend on its secondary or tertiary structure, which is difficult to capture through simple sequence analysis. Therefore, it is necessary to validate the computational predictions with experimental evidence. Based on the results presented above, it can be concluded that in silico proteolysis simulations are valuable tools for guiding the selection of enzymes or enzyme combinations. This approach can be used to optimize the hydrolysis of CGM protein and facilitate the production of cyclic peptides with antioxidant activities.

### 3.2. Purification and Identification of Antioxidant Cyclic Peptide from Hydrolysate

#### 3.2.1. Silica Gel Column Separation and Purification

**Figure 3:**
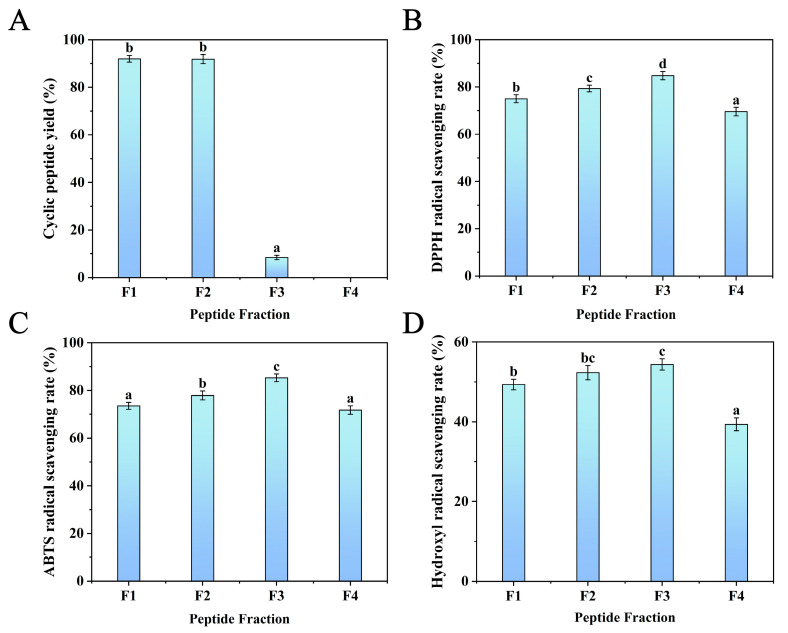
(**A**) Cyclic peptide yield, (**B**) DPPH, (**C**) ABTS, and (**D**) hydroxyl radical scavenging rates of the fractions separated by silica column. Different lowercase letters (a–d) on the bars mean significant difference (*p* < 0.05).

Silica gel chromatography was used to separate the protein hydrolysate [33], which was treated with the optimum enzyme combination (papain+subtilisin). Gradient elution was carried out using chloroform–methanol solutions with the following ratios: 8:2, 7:3, 6:4, and 5:5 (*v/v*). Each eluent (F1, F2, F3, and F4) was collected using the TLC method to determine the cyclic peptide yield. It can be seen from Figure 3A that the F1 and F2 fractions exhibited relatively higher cyclic peptide yields, reaching (91.98 ± 1.43)% and (91.84 ± 1.90)%, respectively, while the cyclic peptide yields of F3 and F4 were very low. Compared with linear peptides and proteins, cyclic peptides have lower polarity and relatively stronger hydrophobicity, and cyclic peptides have higher solubility in low-polarity mobile phases. Therefore, cyclic peptides are eluted earlier than linear peptides and proteins in the mobile phases of 8:2 and 7:3 (*v/v*) chloroform–methanol solvents.

In addition, the antioxidant activity of the four fractions was evaluated by the scavenging ability of DPPH, ABTS, and hydroxyl radicals. As shown in Figure 3B–D, the results show that F3 had the most significant scavenging rates of DPPH (84.82 ± 1.77%), ABTS (85.31 ± 1.62%), and hydroxyl radicals (54.36 ± 1.42%). This might be because the linear peptides in F3 had higher hydrophobicity at the terminal region of the peptide sequence. It was reported that hydrophobic amino acids at the N- and C-terminals could enhance peptide antioxidant activity [31]. Furthermore, F1 and F2 also showed higher scavenging activities of DPPH (74.98 ± 1.67%, 79.31 ± 1.43%), ABTS (73.52 ± 1.43%, 77.88 ± 1.90%), and hydroxyl radicals (49.34 ± 1.32%, 52.32 ± 1.76%). F4 has the lowest scavenging ability for DPPH, ABTS, and hydroxyl radicals. Thus, F1 and F2, which exhibited the highest yield of cyclic peptides and higher DPPH, ABTS, and hydroxyl scavenging rates, were purified using semi-preparative RP-HPLC.

#### 3.2.2. Separation and Purification by Semi-Preparative RP-HPLC

**Figure 4:**
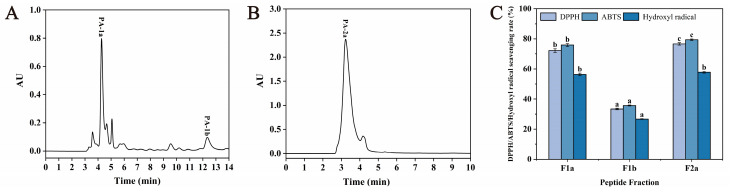
Separation profiles of Fl (**A**) and F2 (**B**) on semi-preparative RP-HPLC chromatography and the DPPH, ABTS, and hydroxyl radical scavenging rates of each subfraction (**C**). Different lowercase letters (a–c) on the bars mean significant difference (*p* < 0.05).

As shown in Figure 4, three main subfractions, including F1a, F1b, and F2a, were isolated from F1 and F2, and the DPPH, ABTS, and hydroxyl radical scavenging activities of these three subfractions were recorded. The results showed that F1a and F2a had high DPPH (72.24 ± 1.15%, 76.63 ± 0.83%), ABTS (75.86 ± 1.01%, 79.41 ± 0.51%), and hydroxyl radical (56.33 ± 0.83%, 57.78 ± 0.61%) scavenging activities. However, F1b showed poor antioxidant activity. Based on the above results, F1a and F2a subfractions were collected for mass spectrometry detection.

#### 3.2.3. Sequence Identification of Antioxidant Cyclic Peptides Using UPLC/MS and MS/MS

**Figure 5:**
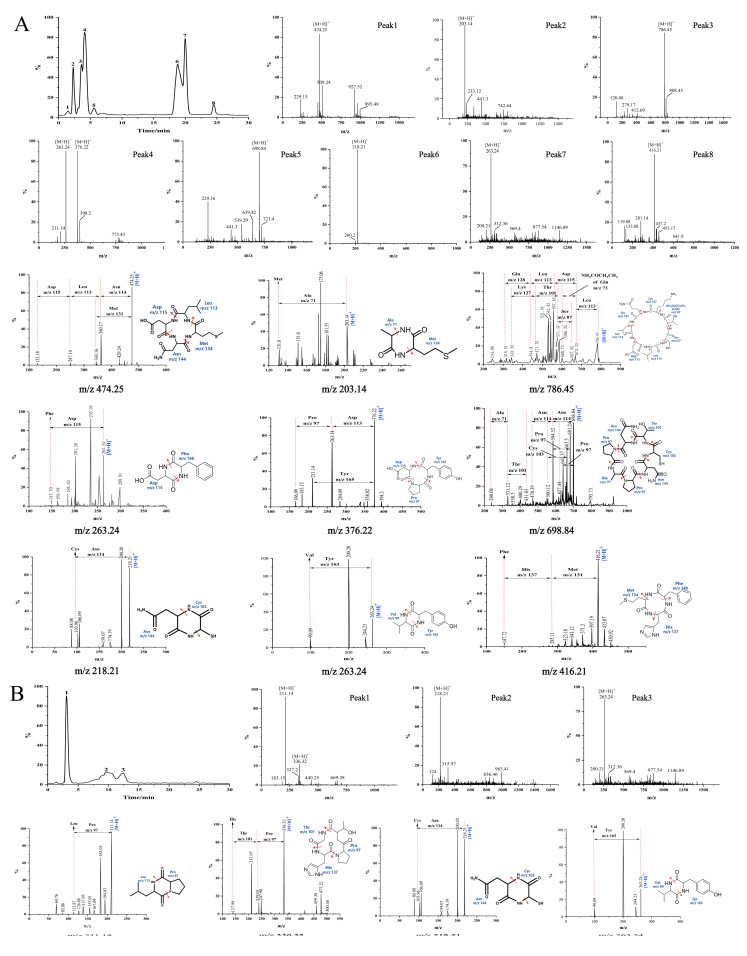
TIC chromatograms, UPLC/MS spectra, and MS/MS spectra of the cyclic peptides in F1a (**A**) and F2a (**B**) and their fragmentation pathway. Peak No.1–8 in the figure represent the peaks detected at different retention times (RT). Arrows show the MS/MS fragmentation positions of the [M+H]^+^ ions.

F1a and F2a had good antioxidant activity, and the applied purification method removed all linear peptides present in these fractions. Thus, these two fractions were subjected to UPLC/MS and MS/MS analysis to determine the sequence of the antioxidant cyclic peptides contained in the fractions. The total current ion chromatograms of Fla and F2a showed eight and three major peaks, respectively (Figure 5). The retention times and protonated molecular ions ([M+H]^+^) detected are summarized in Table 2. The MS/MS spectra of the [M+H]^+^ ions are shown in Figure 5. Collision-induced dissociation (CID) of peptide fragments at high collision energy (kV level) produces “y”- and “b”-series ions, facilitating accurate peptide identification. Based on the “y”- or “b”-series ions in the MS/MS spectra, the m/z values were identified as 474.25 Da, 203.14 Da, 786.45 Da, 263.24 Da, 376.22 Da, 698.84 Da, 218.21 Da, 263.24 Da, 416.21 Da, 211.14 Da, and 336.32 Da. The 11 different peptides of Da are Cyclo(Asp-Leu-Asn-Met), Cyclo(Ala-Met), Cyclo(Gln-Leu-Asp-Leu-Ser-Thr-Lys), Cyclo(Asp-Phe), Cyclo(Pro-Asp-Tyr), Cyclo(Asn-Pro-Ala-Pro-Asn-Cys-Thr), Cyclo(Cys-Asn), Cyclo(Val-Tyr), Cyclo(Met-His-Phe), Cyclo(Leu-Pro), and Cyclo(Pro-His-Thr). The mass difference between adjacent “y”- and “b”-series ions corresponds to the amino acid residue mass; thus, the amino acid sequence of cyclic peptides could be deduced based on complementary “b”- and “y”-series ions. The MS/MS spectra and the fragmentation pathway of these sequences are shown in Figure 5. The hypothesis that the detected peptides are cyclic is usually supported by three facts [43]. The first is that the total molecular mass of the amino-acid residues identified in a peptide is higher by 18 Da than the molecular mass determined experimentally. The second is that the most probable structure modification process is cyclization for peptides composed solely of Leu, Ala, Val, Pro, Gly, Phe, Trp, Ile, and Met residues. The third is that neutral loss of some amino acids was detected in cyclic peptides in some cases. For instance, the [M+H]^+^ of Cyclo(Asp-Leu-Asn-Met) may eliminate Met or Asn. Such neutral loss is unlikely to be observed in linear peptides.

Numerous studies have demonstrated enhanced antioxidant activity by using peptides containing hydrophobic amino acids such as Try, Met, Phe, Val, Ala, Pro, and Leu [44]. This study found that 10 out of the 11 identified cyclic peptides contained one or more hydrophobic amino acids. Among them, six cyclic peptides, including Cyclo(Asn-Leu-Asp-Met), Cyclo(Ala-Met), Cyclo(Leu-Pro), Cyclo(Val-Tyr), Cyclo(Asp-Phe), and Cyclo(Met-His-Phe), had more than 50% of their amino acid residues consisting of hydrophobic amino acids. Peptides containing hydrophobic amino acids enhance free radical scavenging activity by facilitating interactions with lipids or acting as proton or hydrogen donors [45]. In addition, Cyclo(Leu-Pro) has been isolated from the fermentation broth of Bacillussp, and it has exhibited strong inhibition effects on the mycelia growth of fungus and aflatoxin production [46]. Cyclo(Asp-Phe) was identified as the major product of l-Phe-α-l-Asp-GlyOH degradation [47]. Other cyclic peptides, including Cyclo(Asp-Leu-Asn-Met), Cyclo(Ala-Met), Cyclo(Gln-Leu-Asp-Leu-Ser-Thr-Lys), Cyclo(Pro-Asp-Tyr), Cyclo(Asn-Pro-Ala-Pro-Asn-Cys-Thr), Cyclo(Cys-Asn), Cyclo(Val-Tyr), Cyclo(Met-His-Phe), Cyclo(Pro-His-Thr), and Cyclo(Asp-Phe), have not been detected before in nature resources.

#### 3.2.4. Identification of the Cyclization Sites of the Antioxidant Cyclic Peptide

The identified linear peptide precursors from the hydrolysate are shown in Table 3. The cyclization sites of the identified antioxidant cyclic peptides can be inferred by comparing the structures of cyclic peptides and their linear peptide precursors. (The peptide structure comparison method is shown in Figure S1). As can be seen from Table 3, the cyclization sites of the cyclic peptides identified are mainly hydrophobic amino acids (such as Pro, Val, Leu, Met, Phe) and small amino acids (such as Ala, Ser). Literature has shown that the presence of small amino acids (such as Ala, Ser, etc.) at the two terminals (C-terminal and N-terminal) of the peptide sequences has been of importance for the cyclization of linear peptides [41]. In addition, some amino acids, such as Leu, Phe, Ala, Met, Tyr, and Ser, that are determined in the cyclization site of cyclic peptides are also the cleavage sites of papain and subtilisin [48].

### 3.3. Molecular Docking with Keap1

Keap1 is a primary regulator of cellular oxidative stress responses. Disruption of the protein–protein interaction (PPI) between Nrf2 and Keap1 is an attractive strategy to achieve antioxidant effects [49]. If small molecule peptides bind to Keap1 and occupy the Keap1–Nrf2 binding site, it may indicate that they can inhibit the Keap1–Nrf2 interaction and activate the human antioxidant pathway. Therefore, the potential antioxidant mechanism of cyclic peptides detected in the protein hydrolysate of CGM was examined by exploring the relationship between Keap1 and the target cyclic peptides through molecular docking analysis. In addition, the TX6 protein (CID: 121488089), which has been reported to be able to activate the Nrf2 pathway, was selected as the control ligand for molecular docking [50].

**Figure 6:**
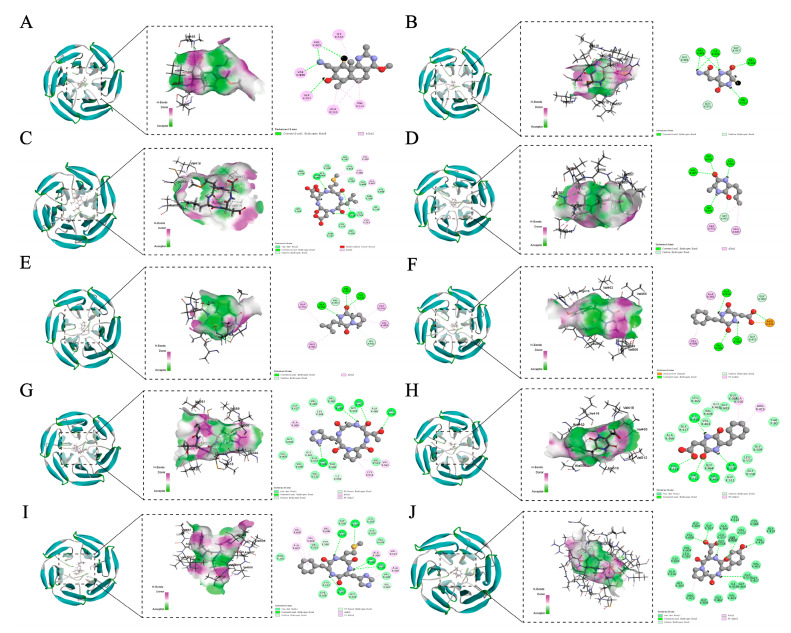
3D and 2D docking diagram of TX6 (**A**), Cyclo(Cys-Asn) (**B**), Cyclo(Asn-Leu-Asp-Met) (**C**), Cyclo(Ala-Met) (**D**), Cyclo(Leu-Pro) (**E**), Cyclo(Val-Tyr) (**F**), Cyclo(Pro-His-Thr) (**G**), Cyclo(Asp-Phe) (**H**), Cyclo(Met-His-Phe) (**I**), and Cyclo(Pro-Asp-Tyr) (**J**) with the active site of Keap1.

The degree of binding of antioxidant peptides to Keap1 mainly depends on the CDOCKER interaction energy, the receptor active site, and the number of interacting amino acid residues. Visualization revealed that the cyclic peptide and Keap1 binding regions were all located near the high-flexibility loop in its Kelch region, which is a key region for the interaction between Keap1 and Nrf2 (Figure 6). A lower CDOCKER interaction energy (IECD) indicates a more stable and stronger binding between the protein and ligand [51]. All cyclic peptides tested in this study showed low docking energy (Table 4). The ligand with the lowest free energy was Cyclo(Pro-Asp-Tyr) (x138.385 kcal/mol), showing the most substantial binding ability to the Keap1. The ligand with the highest free energy was TX6 (−29.7613 kcal/mol), showing the weakest binding ability. It was shown that tested cyclic peptides had better stability and binding affinity than TX6.

In addition, TX6 formed four hydrogen bonds with Val465, Val418, and Gly367 of Keap1 and five hydrophobic interactions with Ile559, Ala366, and Val606 of Keap1 (Table 4). The CDOCKER interaction energy of the tested cyclic peptides with Keap1 ranged from −138.385 to −75.6207 kcal/mol, the number of hydrogen bonds and hydrophobic bonds formed were 4–13 and 2–7, and the binding involved more than 38 amino acid residues (Table 4). Cyclo(Pro-Asp-Tyr) interacted with Gly603, Val604, Val606, Gly605, Gly367, Ala366, Leu557, Val512, Gly559, Val418, Ala510, Ile416, and Gly462 of Keap1 to form 13 hydrogen bonds. In addition, Cyclo(Pro-Asp-Tyr) also formed three hydrophobic bonds with Ala366, Val418, and Arg415 of Keap1. Meanwhile, Cyclo(Ala-Met) showed the fewest hydrogen bonds and hydrophobic interactions among all 10 cyclic peptides. Previous studies have revealed that ligands bind to receptors through multiple intermolecular interactions, including van der Waals forces, hydrogen bonding, electrostatic interactions, and hydrophobic interactions. Among these, hydrogen bonds are the strongest. During the docking process, the amino acid structural arrangements of the cyclic peptides, including Cyclo(Cys-Asn), Cyclo(Asn-Leu-Asp-Met), Cyclo(Ala-Met), Cyclo(Leu-Pro), Cyclo(Val-Tyr), Cyclo(Pro-His-Thr), Cyclo(Asp-Phe), Cyclo(Met-His-Phe), and Cyclo(Pro-Asp-Tyr), could stably bind to Keap1 and exert steric hindrance, thereby preventing Keap1 from attaching to Nrf2 and promoting the accumulation of Nrf2 to express antioxidant genes [45].

## 4. Conclusions

This study used a multidisciplinary approach to isolate and characterize antioxidant cyclopeptides from corn gluten meal (CGM). Computer simulation predicted that cyclopeptides with antioxidant activity could be produced from corn gluten meal (CGM) using a combination of multiple enzymes. Among them, papain+subtilisin was the best solution, and the conclusion of virtual enzymatic hydrolysis was confirmed by actual verification. Silica gel column chromatography and semi-preparative reversed-phase high-performance liquid chromatography separation technology combined with mass spectrometry identification were used to separate and identify 11 cyclopeptides with antioxidant potential. Among the identified cyclopeptides, nine cyclopeptides were able to bind tightly to the pocket site of Keap1 (CDOCKER interaction energy ranged from −138.385 to −75.6207 kcal/mol), and the cyclization sites were mainly hydrophobic amino acids and small molecule amino acids. These cyclopeptides may enhance antioxidant activity by regulating the Keap1–Nrf2 signaling pathway. Although bioinformatics methods may not be able to detect all cyclopeptides with antioxidant potential, this method shows great potential in the discovery of bioactive cyclopeptides because it saves time and cost compared with traditional methods. Therefore, the methods and findings of this study provide valuable insights that can significantly promote the discovery and optimization of new bioactive cyclic peptides from natural protein sources. There is still room for further research on the antioxidant activity of corn cyclic peptides. This paper only measured the antioxidant activity in vitro, and the corresponding cell and animal experiments will be further designed in the future. In addition, corn antioxidant cyclic peptides can be used as resources for developing nutritional health products and cyclic peptide drugs.

## Figures and Tables

**Table 1:** Hydrolysis parameters for each single enzyme and compound-enzyme hydrolysis.

Enzyme	Enzyme Addition (U/g)	pH	Time (h)	Temperature (℃)
Single-enzyme hydrolysis				
Papain	5000	6.5	8	65
Stem bromelain	5000	6.8	8	50
Subtilisin	5000	7.5	8	60
Compound-enzyme hydrolysis (added in a 1:1 ratio)				
Papain+subtilisin	2500 + 2500	7.0	8	63
Stem bromelain+subtilisin	2500 + 2500	7.2	8	55
Papain+stem bromelain	2500 + 2500	6.7	8	58

**Table 2:** Identification data for the antioxidant cyclic peptides using UPLC/MS and MS/MS.

Fraction	Peak No.	RT (min)	Cyclic Peptide	Parent Ions ([M+H]^+^) m/z	Theoretical Mw (Da)	MS/MS
F1a	1	2.295	Cyclo(Asp-Leu-Asn-Met)	474.25	473.56	360.17, 343.16, 247.14, 132.18
	2	3.012	Cyclo(Ala-Met)	203.14	202.29	131.00
	3	3.634	Cyclo(Gln-Leu-Asp-Leu-Ser-Thr-Lys)	786.45	785.91	673.33, 657.82, 600.73, 571.47, 557.46, 471.75, 444.90, 343.35, 315.51
	**4**	**4.170**	**Cyclo(Asp-Phe)**	**263.24**	**262.28**	**147.70**
	**4**	**4.170**	**Cyclo(Pro-Asp-Tyr)**	**376.22**	**375.40**	**263.14, 166.09**
	5	5.527	Cyclo(Asn-Pro-Ala-Pro-Asn-Cys-Thr)	698.84	697.78	681.24, 663.30, 641.37, 584.52, 560.12, 470.39, 431.03, 331.12, 240.08
	**6**	**18.868**	**Cyclo(Cys-Asn)**	**218.21**	**217.26**	**103.96**
	**7**	**19.963**	**Cyclo(Val-Tyr)**	**263.24**	**262.32**	**99.09**
	8	24.458	Cyclo(Met-His-Phe)	416.21	415.53	285.11, 147.72
F2a	**1**	**2.994**	**Cyclo(Leu-Pro)**	**211.14**	**210.29**	**113.87**
	**1**	**2.994**	**Cyclo(Pro-His-Thr)**	**336.32**	**335.38**	**239.01, 137.99**
	2	9.906	Cyclo(Cys-Asn)	218.21	217.26	103.96
	3	12.335	Cyclo(Val-Tyr)	263.24	262.32	99.09

**Table 3:** Linear peptide precursors of the cyclic peptides identified from the hydrolysate.

Sequence of Linear Peptide Precursors	Molecular Weight (Da)	Sequence of Cyclic Peptide	Molecular Weight (Da)	Cyclization Site
Asn-Leu-Asp-Met	493.84	Cyclo(Asp-Leu-Asn-Met)	474.25	Asn, Met
Ala-Met	221.74	Cyclo(Ala-Met)	203.14	Ala, Met
Ser-Thr-Lys-Gln-Leu-Asp-Leu	804.12	Cyclo(Gln-Leu-Asp-Leu-Ser-Thr-Lys)	786.45	Ser, Leu
Asp-Phe	281.87	Cyclo(Asp-Phe)	263.24	Asp, Phe
Pro-Asp-Tyr	394.82	Cyclo(Pro-Asp-Tyr)	376.22	Pro, Tyr
Ala-Pro-Asn-Cys-Thr-Asn-Pro	716.99	Cyclo(Asn-Pro-Ala-Pro-Asn-Cys-Thr)	698.84	Ala, Pro
Cys-Asn	236.69	Cyclo(Cys-Asn)	218.21	Cys, Asn
Val-Tyr	281.79	Cyclo(Val-Tyr)	263.24	Val, Tyr
His-Phe-Met	434.63	Cyclo(Met-His-Phe)	416.21	His, Met
Leu-Pro	229.72	Cyclo(Leu-Pro)	211.14	Leu, Pro
Pro-His-Thr	354.79	Cyclo(Pro-His-Thr)	336.32	Pro, Thr

**Table 4:** Docking energy, number of hydrogen bonds, and hydrophobic interactions and interaction sites of cyclic peptides with Keap1.

No.	Ligand	IECD (kcal/mol)	Hydrogen Bond		Hydrophobic Bond	
			Number	Amino Acid Residues	Number	Amino Acid Residues
1	TX6	−29.7613	4	Val465, Val418, Gly367	5	Ile559, Ala366, Val606
2	Cyclo(Cys-Asn)	−75.6207	10	Gly462, Val463, Ile416, Gly417, Val418, Val512, Gly511	–	–
3	Cyclo(Asn-Leu-Asp-Met)	−79.0846	4	Val467, Val514	4	Val418, Cys368, Ala607, Cys513
4	Cyclo(Ala-Met)	−79.3505	5	Val465, Val512, Ala510, Ile416, Gly462	2	Ala556, Arg415
5	Cyclo(Leu-Pro)	−84.7523	6	Ala510, Val463, Val465, Val512, Ile416	5	Ala556, Arg415, Val418, Ala366
6	Cyclo(Val-Tyr)	−107.268	6	Ala510, Gly462, Gly417, Ile416, Val465	2	Ala366, Val606
7	Cyclo(Pro-His-Thr)	−111.776	11	Cys368, Val418, Val467, Ala466, Val420, Ile559, Val606, Gly367	4	Ala366, Val606, Cys513, Val561
8	Cyclo(Asp-Phe)	−113.424	6	Ile416, Gly462, Ala510, Val465, Val512, Val418	2	Ala556, Arg415
9	Cyclo(Met-His-Phe)	−121.817	6	Thr560, Val512, Ile559, Val465, Val418, Val467, Gly367	7	Ala607, Val608, Val369, Val606, Ala366, Val517, Ala466
10	Cyclo(Pro-Asp-Tyr)	−138.385	13	Gly603, Val604, Val606, Gly605, Gly367, Ala366, Leu557, Val512, Gly559, Val418, Ala510, Ile416, Gly462	3	Ala366, Val418, Arg415

## Data Availability

The original contributions presented in the study are included in the article/Supplementary Material, further inquiries can be directed to the corresponding authors.

## Supplementary Materials

The following supporting information can be downloaded at: https://www.mdpi.com/article/10.3390/foods14101709/s1; Figure S1. Comparative methods for cyclic peptide structures.

## Abbreviations

The following abbreviations are used in this manuscript:

CGM	Corn gluten meal
Keap1	Kelch-like ECH-associated protein 1
Nrf2	Nuclear factor erythroid 2-related factor 2
