# Supplementary material for: Bioinformatics-Assisted Discovery of Antioxidant Cyclic Peptides from Corn Gluten Meal
Source: Foods. 2025 May 12;14(10):1709. doi: 10.3390/foods14101709 (PMC12111382; doi:10.3390/foods14101709)
Supplement: Supplementary file 1 [file foods-14-01709-s001.zip › foods-3603481-supplementary.pdf]

Supplementary Documents

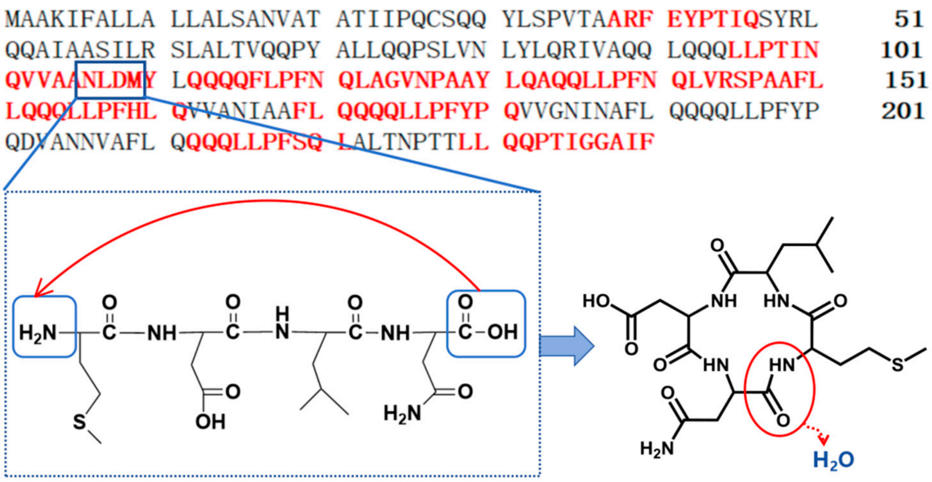

Figure S1. Comparative methods for cyclic peptide structures
